# Behind a Voice There is a Speaker: Why Vocal Emotion Research Needs to Become ‘Personal’

**DOI:** 10.1007/s42761-025-00317-w

**Published:** 2025-07-04

**Authors:** Ana P. Pinheiro

**Affiliations:** Faculdade de Psicologia, CICPSI, Universidade de Lisboa, Lisbon, Portugal

**Keywords:** Voice, Emotion, Speaker identity, Person characteristics, Time course

## Abstract

The voice is a powerful social signal and a primary channel for communicating emotions when speakers are out of view. When we hear an emotional voice, we quickly form an impression of the person behind it. Neurocognitive models emphasize the multi-step dynamic operations that occur when listeners decode emotional information from vocal sounds. However, these models have primarily focused on stimulus quality, often neglecting the perception of other relevant person characteristics (e.g., gender, age, personal identity) evolving on different timescales. How do the emerging details about the speaker affect how listeners decode emotional information? And how are these different types of information integrated into a comprehensive impression of the speaker? This review examines recent data highlighting multiple stages of vocal expression analysis and the interplay between distinct types of nonverbal information revealed in the speaker’s voice. It serves as a starting point for broader research examining how distinct person characteristics, perceived simultaneously or in close succession, interact and affect the decoding of vocal emotions.

*“And Vincent sadly recalls an old idea of his: people always think that a man’s fortunes are more or less determined by his appearance, by the beauty or ugliness of his face, by his size, by his hair or lack of it. Wrong. It is the voice that decides it all.*”

(Milan Kundera, ‘Slowness’, p. 60).

The voice is one of our most powerful instruments: it is fundamental for communication and the dominant mode for social engagement across different cultures (Scott, 2019). Specifically, it is a rich source of emotional information that can be communicated even when speakers are out of view (e.g., on the telephone or over the radio) or without the benefit of daylight. It is therefore considered ‘special’ (Frühholz & Belin, 2018). The study of vocal emotions has seen renewed interest in recent decades. Nevertheless, far less is known about the processing of voices than faces (e.g., Schirmer & Adolphs, 2017). This discrepancy may be due to the intrinsic temporal dimension of sounds, which makes the study of voices more challenging.

There is a relative consensus that the perception of vocal emotions – including nonverbal vocalizations and emotional prosody – involves distinct processing stages. Neurocognitive models (e.g., three-step; Schirmer & Kotz, 2006) have provided insights into the operations involved in extracting emotional meaning from voices and their underlying neural mechanisms. Time-sensitive methods such as electroencephalography (EEG) have proven especially useful for elucidating the relative time course of these operations, which depend on the continuous monitoring of dynamically changing acoustic cues. EEG studies support three consecutive phases of vocal emotional decoding (Castiajo & Pinheiro, 2021; Conde et al., 2022; Liu et al., 2012; Paulmann & Kotz, 2008; Pell et al., 2015). The analysis of vocal emotional expressions starts with the sensory encoding of relevant acoustic cues (~ 100 ms post-voice onset). This is followed by rapid salience detection (~ 200 ms). The third stage involves the cognitive evaluation of the emotional significance of the voice (~ > 300 ms), leading to the refinement of emotional stimulus representations (Giordano et al., 2021).

However, behind a voice there is always a speaker. When hearing a voice, listeners rapidly perceive a wealth of temporally stable person characteristics, including the physical (e.g., age: Bozkurt & Soley, 2022; Hunter et al., 2016; Lavan, 2022; gender: Barreda & Assmann, 2021; Lass et al., 1976; Owren et al., 2007; body size: Gonzalez, 2003; Sell et al., 2010), psychological and social status (e.g., warmth/trustworthiness or dominance: McAleer et al., 2014; Mileva & Lavan, 2023; Pinheiro et al., 2021) of the speaker, i.e., what they might be like as a person. How does this information affect or interact with the perception of vocal emotions, which are dynamically changing social signals? These core aspects of person perception from voices are currently missing from theoretical models of emotional voice perception.

Notably, both person-relevant (Lavan, 2023; Lavan et al., 2024; Mileva & Lavan, 2023) and emotional nonverbal information (Castiajo & Pinheiro, 2019; Liu et al., 2012; Pinheiro, Barros, Dias et al., 2017; Pinheiro, Barros, Vasconcelos et al., 2017; Pinheiro, Barros et al., 2016; Pinheiro, Rezaii et al., 2016) can be decoded in less than a second of exposure to a person’s voice. The recognition of both speaker identity and vocal emotion partly relies on processing similar acoustic features (see Table 1) such as pitch – the perceptual correlate of fundamental frequency (F0; Titze, 1994) and the most perceptually salient non-verbal vocal feature, with clear evolutionary substrates (e.g., Aung & Puts, 2020; Puts et al., 2016). F0 also forms a major cue in the perception of other person-related characteristics, including physical (e.g., gender, age), psychological (e.g., personality), and social attributes (e.g., social rank) of the speaker (O’Connor & Barclay, 2017; Skuk & Schweinberger, 2014). For example, as human voice pitch is sexually dimorphic, it serves as a salient cue for gender perception: mean voice pitch is typically substantially lower in male (~ 120 Hz) than in female (~ 220 Hz) voices (Childers & Wu, 1991). Nevertheless, the relevance of pitch does not negate the role of other acoustic cues in nonverbal voice perception (e.g., formant frequencies in speaker recognition; Latinus & Belin, 2012).

**Table 1:** Similarities and differences between identity recognition and emotion recognition through voices

	Emotion	Identity
**Mean accuracy**	• Overall^a^: 84.3% (Laukka & Elfenbein, 2021) • Positive emotions^b^: 79.7% (Laukka & Elfenbein, 2021) • Negative emotions^c^: 84.9% (Laukka & Elfenbein, 2021)	• Unfamiliar voice recognition: 89% (Stevenage et al., 2024) • Famous voice recognition: 23% (Jenkins et al., 2021); 49% (Stevenage et al., 2024)
**Acoustic cues**	• F0, speech rate (e.g., Juslin & Laukka, 2003)	• F0, formants (e.g., Baumann & Belin, 2010) *But the weighting of distinct acoustic cues may depend on the listener (e.g., Lavan, Kreitewolf et al., 2021; Lavan, Mileva et al., 2021)
**Effects on other cognitive functions**	• Attention (e.g., Pinheiro, Barros, Dias et al., 2017; Pinheiro, Barros, Vasconcelos et al., 2017; Pinheiro, Barros et al., 2016; Pinheiro, Rezaii et al., 2016) • Memory (e.g., Pichora-Fuller et al., 2016; Schirmer, 2010) • Speech comprehension (e.g., Mauchand et al., 2021; Pell et al., 2011; J. P. Walker et al., 2001; Yi et al., 2020) • Spontaneous trait inference (e.g., Pinheiro et al., 2021)	• Attention (e.g., Conde et al., 2016, 2018; Pichora-Fuller et al., 2016) • Memory (e.g., Sheffert & Fowler, 1995; Zäske et al., 2018) • Speech comprehension (e.g., Foucart et al., 2015; Van Berkum et al., 2008) • Spontaneous trait inference (e.g., Jiang et al., 2019)
Neural correlates*	• Right anterior STS (e.g., Frühholz & Ceravolo, 2018) • Amygdala (e.g., Frühholz & Ceravolo, 2018) • Inferior frontal cortex (e.g., Frühholz & Ceravolo, 2018)	• Right anterior STS (e.g., Schweinberger & Zäske, 2018) • Inferior frontal cortex (e.g., Schweinberger & Zäske, 2018)
**Functional lateralization**	Right (e.g., Frühholz & Ceravolo, 2018)	Right (e.g., Schweinberger & Zäske, 2018)
**Maturation**	• Early sensitivity to the emotional relevance of voices (37 weeks gestational age: Hou et al., 2024; newborns: Mastropieri & Turkewitz, 1999) • Early functional specialization of emotional voice processing (newborns: Cheng et al., 2012) • Protracted maturation of vocal emotion recognition in adolescence (Morningstar et al., 2018, 2020)	• Early recognition of mother’s voice (third trimester of pregnancy: e.g., Kisilevsky et al., 2003) • Early preference for mother’s voice (newborns: Abrams et al., 2016; Purhonen et al., 2004) • Protracted maturation of speaker identity recognition in adolescence; performance dip in early adolescence (Mann et al., 1979)

*Core regions of a large-scale brain network. STS: superior temporal sulcus. ^a^Overall recognition accuracy is based on all included emotion categories in each respective study in the meta-analysis. ^b^Positive emotions included in the meta-analysis were: achievement, amusement, awe, calm, contentment, desire, happiness, interest, love, lust, pride, relief, and sympathy. ^c^Negative emotions included in the meta-analysis were: anger, contempt, doubt, disgust, embarrassment, fear, pain, sadness, and shame

While the processing of speech-related linguistic features has been predominantly attributed to the left hemisphere (‘what’), the right hemisphere is largely specialized in processing speaker-related paralinguistic features (‘who’ and ‘how’; Belin et al., 2004; Formisano et al., 2008; Morillon et al., 2022) relevant for speaker identity and emotion recognition. In particular, voice processing areas in the right superior temporal sulcus/gyrus are not only sensitive to speaker identity or vocal emotions (Frühholz & Grandjean, 2013), but also to other person-related features such as gender (Charest et al., 2013; Junger et al., 2013; Lattner et al., 2005), supporting the relative right hemispheric preference for the perception of pitch variation (Johnsrude et al., 2000).

Based on experimental, clinical, and neurophysiological evidence, it has been proposed that the identity and emotion dimensions of the voice are partially dissociable (Belin et al., 2004; Garrido et al., 2009; Spreckelmeyer et al., 2009) but interact with each other (Belin et al., 2004)*.* Following a low-level auditory analysis and a structural analysis of vocal sounds, three functionally independent but partially interacting pathways support the perception of speech, emotion, and identity information (Belin et al., 2004). However, while there is evidence for interactions between speech and speaker perception, less is known about how nonverbal vocal signals such as speaker identity and emotion interact in time, space, and function.

In the following sections, the time course of voice-specific information processing is described. Then, examples are provided that illustrate the important interactions between emotion processing and speaker-relevant features. These examples suggest that the field of vocal emotion research could advance by becoming more ‘personal’.

## The Time Course of Voice Perception

As voices are inherently dynamic, voice perception is dependent on the integration of acoustic information in time. A rapid discrimination of voices from other auditory object categories (e.g., birdsong, environmental sounds) was documented within 150 ms post-stimulus onset (Charest et al., 2009; Schirmer & Gunter, 2017).

**Fig. 1:**
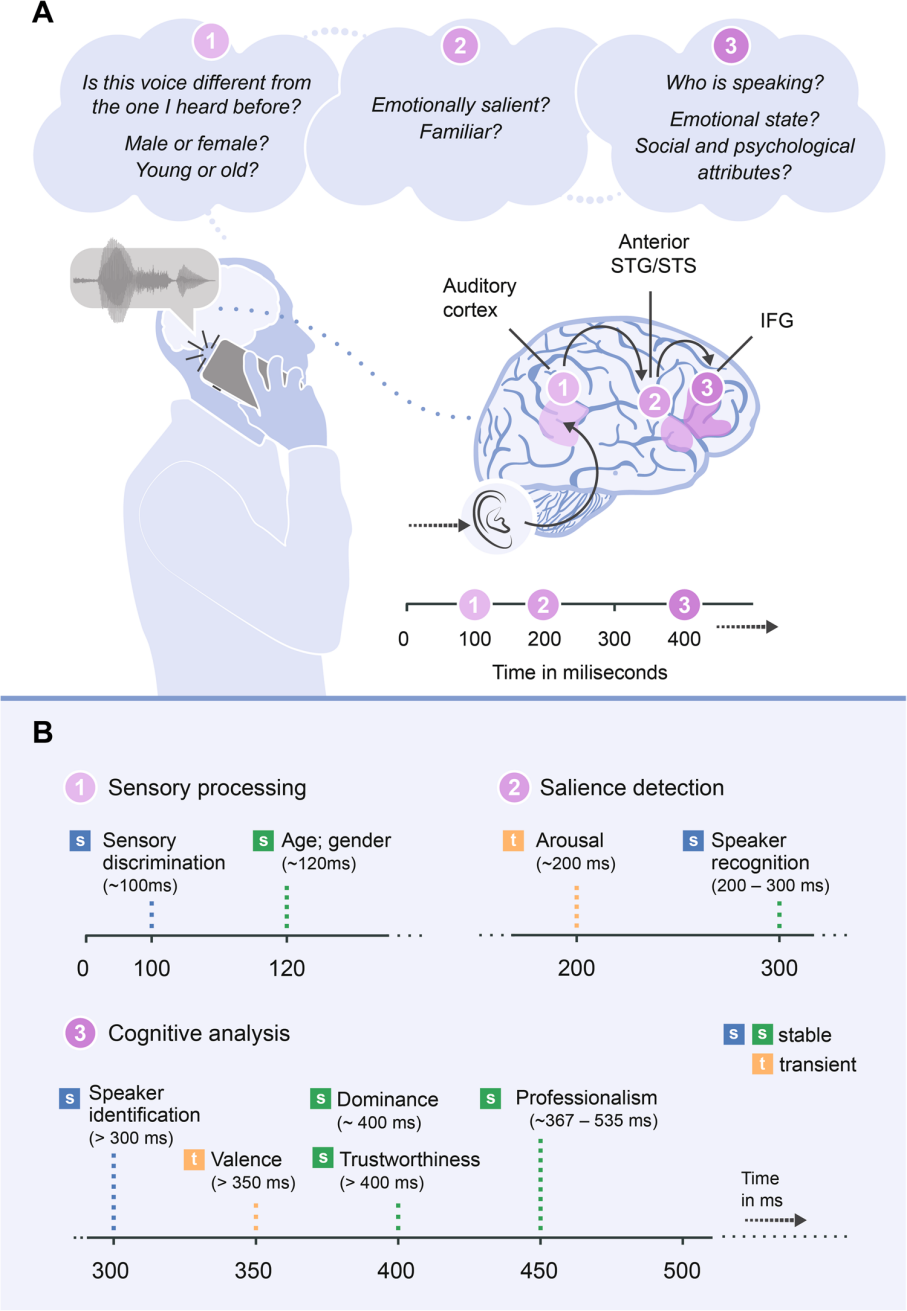
The time course of nonverbal voice perception. A. Three stages of vocal expression analysis are highlighted and their neural correlates are illustrated (e.g., Schirmer & Kotz, 2006; for simplification, only cortical brain regions are shown). B. The putative evolution of brain responses reflecting activation of representations related to emotion, identity, and person characteristics. Neuroimaging findings support a multistep model of voice identity (Bestelmeyer & Mühl, 2022) and emotion (Conde et al., 2022) perception. More recent findings (Lavan et al., 2024) also highlight a staggered time course of person perception from voices, with physical characteristics being decoded earlier (from ~ 120 ms) than psychological and social characteristics (~ 360 ms onward). The time course of these representations may fit into distinct stages of nonverbal voice processing, according to which early representations are more strongly shaped by voice acoustics (sensory processing stage), whereas later processing stages (reflected in protracted ERP components, such as the LPP) deal with higher-level representations of voice identity and vocal emotion, as well as with more abstract speaker-related characteristics (e.g., social and psychological attributes). Based on the existing evidence, a strong possibility – to be tested in future studies – is that all these types of information are processed in parallel, although some may take longer to be processed (e.g., social traits). An integrative representation is then generated, representing the product of the simultaneous interactions among all of them. *Note.* Examples of references: (1) Emotion: Arousal (Liu et al., 2012); Valence (Pinheiro, Barros, Dias et al., 2017; Pinheiro, Barros, Vasconcelos et al., 2017). (2) Identity: Speaker identity (Iannotti et al., 2022; Plante-Hébert et al., 2021; Schweinberger et al., 2011; Spreckelmeyer et al., 2009; Zäske et al., 2014); Acoustic *vs.* cognitive representations of speaker identity (Bestelmeyer & Mühl, 2022); Personal relevance of the speaker (Graux et al., 2013; Pinheiro et al., 2023; Pinheiro, Barros et al., 2016; Pinheiro, Rezaii et al., 2016). (3) Person-related features: Age (Lavan et al., 2024); Gender (Latinus & Taylor, 2012; Lavan et al., 2024; Paulmann & Kotz, 2008); Dominance (Lavan et al., 2024); Professionalism (Lavan et al., 2024); Trustworthiness (Lavan et al., 2024)

EEG studies may inform on how distinct impressions emerge while listening to a voice, from stimulus onset onward, independently of postperceptual judgements. The time course of voice perception (see Fig. 1) can be investigated by examining modulations of evoked potentials such as the N1 (at ~ 100 ms post-voice onset), P2 (at ~ 200 ms), and Late Positive Potential (LPP; after ~ 500 ms).

Emotional information is available within 200 ms post-voice onset and may alter the sensory processing of vocal input. Emotional vocalizations are rapidly differentiated from neutral vocalizations in temporal cortical areas (Liu et al., 2012; Paulmann & Kotz, 2008; Pell et al., 2015; Sauter & Eimer, 2010), shown in P2 amplitude modulations. These effects may arise even earlier for nonverbal vocalizations compared to speech prosody (Liu et al., 2012; Pell et al., 2015). These findings align with behavioral data suggesting that acoustic modulations occurring within the first 300 ms after stimulus onset represent the most critical time window for explicit identification of emotions from nonverbal vocalizations (Castiajo & Pinheiro, 2019). This salience detection stage marks the decoding of motivationally significant features of the voice (Pell & Kotz, 2021). A finer discrimination of emotional vocalizations as a function of valence occurs later, after 300 ms post-stimulus onset (Pinheiro, Barros, Dias et al., 2017; Pinheiro, Barros, Vasconcelos et al., 2017), with some emotions being recognized faster than others (e.g., anger may be recognized faster than happiness in pseudosentences; Pell & Kotz, 2011). As vocal emotion expressions have unique recognition points over time (Castiajo & Pinheiro, 2019; Pell & Kotz, 2011), these temporal differences could affect later processing stages indexing the cognitive analysis of vocal expressions (e.g., Late Positive Potential), when the mental representation of what is being communicated is refined (Kotz & Paulmann, 2011).

The processing of speaker identity also starts early, with speaker discrimination, recognition, and identification producing distinct effects. Speaker discrimination (e.g., detecting whether two voices belong to the same speaker or not) occurs very early in voice perception, within the first 100 ms post-stimulus onset when familiar voices are presented (e.g., Graux et al., 2015). Speaker identity recognition – or the feeling of having heard a given voice before – begins around 200–300 ms for personally familiar voices (e.g., romantic partners, known lecturers; Schweinberger et al., 2011) and laboratory-trained or newly learned voices (Plante-Hébert et al., 2021; Zäske et al., 2014). However, the personal relevance of the speaker (i.e., my voice *vs.* the voice of an unfamiliar speaker) could produce even earlier effects, around 100 ms after voice onset (Conde et al., 2018; Pinheiro et al., 2023; Pinheiro, Barros et al., 2016; Pinheiro, Rezaii et al., 2016). Later effects (> 500 ms) may reflect activation of episodic or semantic memory of a speaker, associated with voice identification processes, or knowing who is speaking (Plante-Hébert et al., 2021).

Listeners also form initial impressions of physical, psychological, and social characteristics of speakers within 400 ms of voice exposure (Lavan, 2023; Lavan et al., 2024; Mileva & Lavan, 2023). That is, person perception starts very early in auditory processing. Although person characteristics (e.g., age, gender, health) are highly inter-correlated (Lavan et al., 2024; Scott, 2008), recent studies support a temporal hierarchy in person perception from voices (Lavan et al., 2024). This suggests that person-relevant characteristics are not perceived holistically at the same time (Lavan et al., 2024). Specifically, the processing of person-general characteristics such as speaker’s age or gender starts earlier than the processing of trait and social characteristics, within 100 ms post-voice onset (Lavan et al., 2024). The rapid decoding of some physical characteristics of the speaker could potentially provide an early bias for emotional salience detection and decoding (discussed in the Section “Other person-general characteristics”). Impressions of a person’s social (e.g., level of education, poshness, professionalism) and trait (e.g., trustworthiness, attractiveness, dominance) characteristics emerge later, after 350 ms post-stimulus onset. These impressions can contribute to a more fine-grained representation of vocal information, co-occurring with enhanced elaborative processing of the emotional significance of the voice or with activation of more detailed information about a familiar speaker, typically reflected in modulations of late potentials that are invariant to voice acoustics (e.g., Late Positive Potential; Hajcak & Foti, 2020). Together, these findings confirm that voice perception unfolds over time for a number of person characteristics.

## Speaker Identity and Familiarity

Vocal emotion research can become more ‘personal’ by exploring how factors like the listener’s familiarity with the speaker’s unique identity affect vocal emotional decoding. Few EEG studies have directly examined the interaction between speaker identity and emotion. Some findings suggest that the recognition of emotional meaning may occur earlier (~ 200 ms) than (unfamiliar) speaker identity discrimination (~ 300 ms; Spreckelmeyer et al., 2009). This supports the idea that emotional cues are prioritized in voice processing over other vocal features (e.g., spatial location: Pinheiro et al., 2019; Temudo & Pinheiro, 2025). These temporal differences could explain why vocal emotions can facilitate speaker identity learning in behavioral tasks, i.e., how new voices become familiar (Kim et al., 2019). However, this facilitation does not occur when vocal emotions differ during encoding and testing (Xu & Armony, 2021) or when emotional vocalizations are produced under reduced volitional control (e.g., spontaneous laughter; Lavan et al., 2016).

Most studies have nevertheless focused on unfamiliar speakers. Familiarity effects were reported ~ 100 ms post-stimulus onset for personally relevant voices (Conde et al., 2018; Graux et al., 2013; Pinheiro et al., 2023; Pinheiro, Barros et al., 2016; Pinheiro, Rezaii et al., 2016), suggesting that speaker familiarity could influence the analysis of vocal emotional expressions before the salience detection stage (~ 200 ms post-stimulus onset). Distinct mechanisms have been proposed to account for familiar *vs.* unfamiliar voice processing: whereas the recognition of familiar voices relies on holistic processing, the processing of unfamiliar voices is more dependent on a feature-based perceptual mode (Kreiman & Sidtis, 2011). Increased experience with a specific speaker’s voice – whether because they are famous, personally known, or introduced in an experimental paradigm (Lavan & McGettigan, 2023) – is known to facilitate voice identity recognition. Similar effects could be observed for emotion recognition, as familiar listeners have access to person-specific representations that likely include information on voice variability (Lavan et al., 2019). Accordingly, there is an accuracy advantage in emotion recognition when emotions are produced in the (more familiar and personally relevant) self-voice compared to an unfamiliar voice (Rachman et al., 2019), in one’s own native language compared to a foreign language (Nakai et al., 2023), or by in-group speakers (Laukka & Elfenbein, 2021; Sauter et al., 2010).

Specifically, personally valued voices are richer and more relevant signals than unfamiliar voices (Lavan & McGettigan, 2023), and are perceived as more emotionally intense (Stoop et al., 2020). As emotion and familiarity are linked (Baudouin et al., 2000; Gerard et al., 1973; Van Lancker & Ohnesorge, 2002; Zajonc, 1968), emotional salience could be enhanced for vocal emotions produced by a personally familiar speaker. This hypothesis is supported by the observation that personally familiar voices engage additional systems associated with socio-emotional processing, including the amygdala and reward-related regions (e.g., ventromedial prefrontal cortex; nucleus accumbens; Abrams et al., 2016), likely providing a neurobiological basis to the colloquial expression ‘it’s so good to hear your voice’ (McGettigan, 2015).

**Fig. 2:**
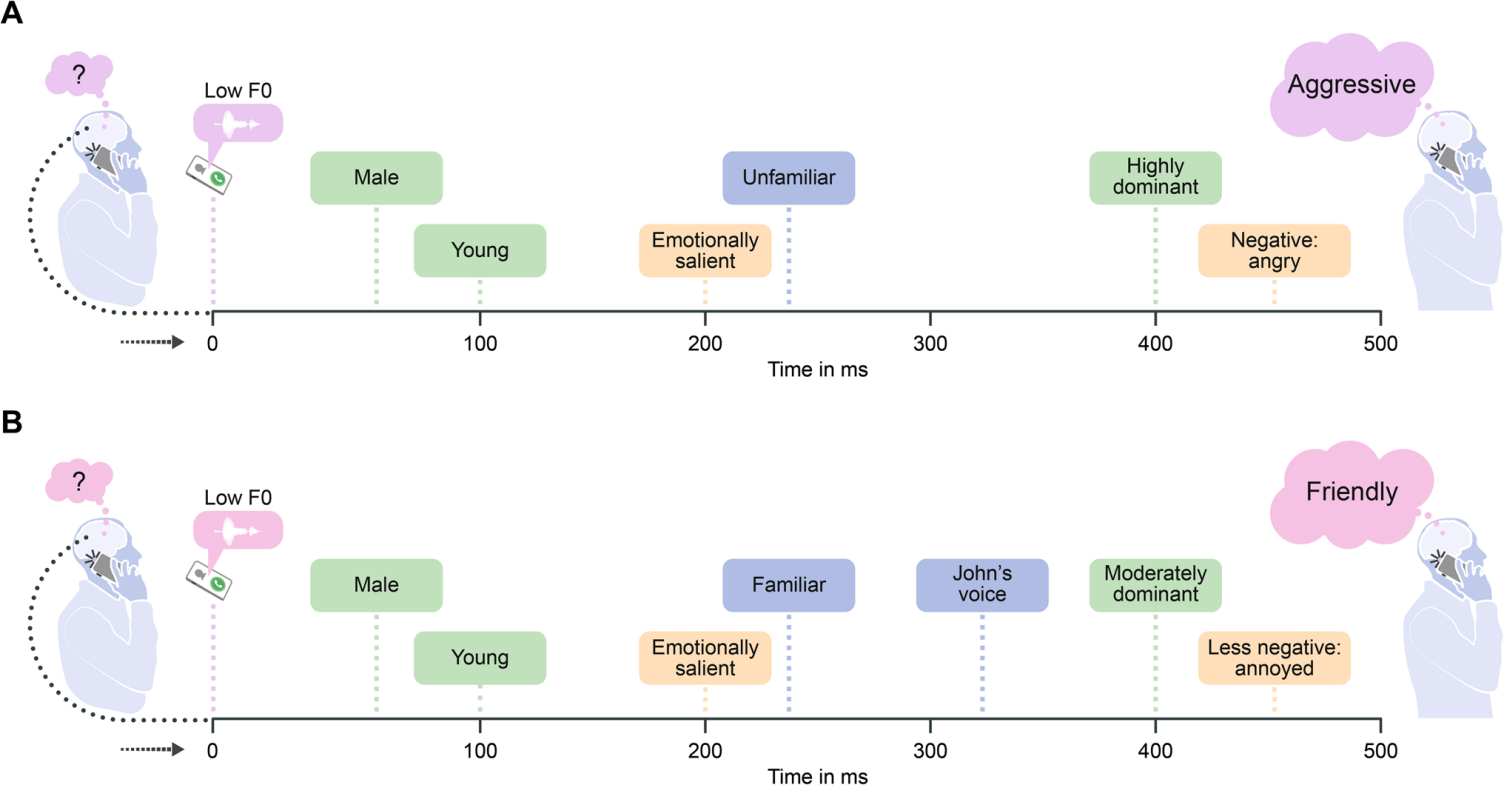
Interactions between emotion, person characteristics, and identity in voice perception: two examples. Panel A considers a situation in which a listener hears an emotionally ambiguous expression from an unfamiliar speaker. During early stages of voice processing, multiple interpretations are possible. The perception of the physical features of the speaker is very rapid. As information accumulates, representations become more fine-grained and impressions may be updated over time. This is especially relevant in the case of voices due to the inherently dynamic nature of vocal cues. In particular, acoustic cues (e.g., low F0) may activate categories (e.g., male) that in turn activate stereotypes (e.g., aggressive) during perception. For example, the category ‘male’ shares conceptual associations with larger body size, physical strength, and dominance, and therefore with hostility and threat. As such, this could increase the probability of anger judgements. On the contrary, higher-pitched (i.e., feminine-sounding) voices may be perceived as more affiliative (e.g., happier or surprised) than lower-pitched (masculine-sounding) voices. In turn, specific emotions may more likely lead to the activation of certain person representations, at least when no further information about the speaker is available (as is the case with unfamiliar speakers). For example, a person who expresses anger may be perceived as more aggressive than a person who expresses sadness. Thus, emotional expressions may often be conflated with social categories (e.g., see Bijlstra et al., 2014 for evidence from face perception research). This means that in the realm of first impressions, features such as ‘aggressive’ and ‘angry’ may not be perceptually distinguishable. Note that the links between emotion and person characteristics could differ for familiar and unfamiliar speakers. Panel B exemplifies top-down effects of speaker knowledge in the case of a personally familiar voice, for which the listener can additionally access emotionally and socially salient information (e.g., whether one likes the speaker or not; specific biographical knowledge about the speaker). Accumulated knowledge that the speaker is kind, in the context of social interactions, may bias the perception of emotional and person-related information. In this specific example, vocal expressions could be perceived less negatively. Findings from face perception research suggest that dominance and trustworthiness are positively correlated when judging close and admired others, whereas they are negatively correlated when judging unfamiliar others (Cuddy et al., 2009; Kraft-Todd et al., 2017). In the example provided, a moderately dominant familiar speaker could arguably still be perceived as trustworthy

Top-down effects of person knowledge (associated with activation of episodic or semantic memory for a given speaker) could thereby exert top-down effects, modulating both early and late voice processing stages (see, for example, Hassin & Trope, 2000 and Suess et al., 2015 for evidence from face perception research, i.e., “*reading into faces*”). A strong possibility is that negative person-related information (e.g., “speaker X is aggressive”) could, for example, lead to the perception of a speaker’s voice as more negative, compared to a similar vocal expression from a speaker for whom one has relatively neutral biographical information or who is unfamiliar (see Fig. 2 for a related example). This suggests that the person behind the voice could also shape the perception of vocal emotions.

## Other Person-General Characteristics

A second way that vocal emotion research can get more ‘personal’ is by operationalizing a wide range of person characteristics perceived from voices, beyond just personal identity. As noted above, vocal expressions of both familiar and unfamiliar speakers convey information about the speaker’s emotional state as well as adaptive details about specific physical and personality traits, such as age and body size (Anikin et al., 2021; Bruckert et al., 2005; Krauss et al., 2002; Pisanski et al., 2017), masculinity/femininity (Borkowska & Pawlowski, 2011; Cartei et al., 2014), attractiveness (Re et al., 2012), dominance (Puts et al., 2007), formidability (Aung & Puts, 2020), competence (Klofstad et al., 2012), likeability (Parker & Borrie, 2018), or trustworthiness (O’Connor & Barclay, 2017; Parker & Borrie, 2018). These speaker characteristics play an important role in perceptual judgements of an unfamiliar voice or in recognizing a personally familiar voice (Lavan & McGettigan, 2023), and they are likely to be integrated into a representation of the emotion being communicated. Nonetheless, they are often studied separately (Lavan & McGettigan, 2023).

Representations of physical features (age, gender) emerge approximately 120 ms post-voice onset (Lavan et al., 2024; Owren et al., 2007), before salience detection. These early neural responses are best explained by acoustic properties of the stimulus, particularly F0 (Lavan et al., 2024)*.* Representations of trait (e.g., trustworthiness, attractiveness, dominance) and social characteristics (e.g., level of education, poshness, professionalism) emerge after 260 ms post-voice onset (Lavan et al., 2024), following the stage of emotional salience detection. These later responses are better explained by abstracted category-level information (Lowe et al., 2021).

Person characteristics available to perception earlier in time may shape how characteristics that become available later are perceived (see Fig. 2). Social categories such as age and gender may alter how emotion expressions are perceived and what trait impressions are formed, with wide-ranging effects on social interactions. For example, early gender inferences, formed within as little as 25 ms of exposure to voices (Lavan, 2023; Owren et al., 2007), could bias subsequent processing according to gender-emotion stereotypes. This is possible as pitch information (used as an index of gender) is decoded from the voice shortly after stimulus onset (Latinus & Taylor, 2012). For example, low-pitched voices tend to be associated with aggressive intent (Zhang et al., 2021) and threat (Taylor & Reby, 2010), as well as with enhanced perceived physical strength (Raine et al., 2019) or upper body strength – related to fighting ability (Sell et al., 2010). In many animals, low F0 is typically associated with threat-related sounds, whereas higher F0 is associated with more positive sounds (Bartholomew & Collias, 1962; Hoeschele et al., 2010; see also Morton, 1977). Thereby, male voices – characterized by lower F0 than female voices – could be more likely associated with emotions such as anger (an emotion closely associated with aggression) than female voices. Consistently, men who lower (*vs.* raise) their pitch when speaking tend to be perceived as more dominant and threatening (Cheng et al., 2016; Fraccaro et al., 2013). Relatedly, semantic cues with negative valence (e.g., words such as “fraud”, “liar”, or “corrupt”) are perceived even more negatively in the context of low- (*vs.* high-) pitched voices (O’Connor & Barclay, 2018). Gender inferences could additionally fuel inferences of male voices being more dominant, and of female voices being less dominant and more submissive, but also more trustworthy, given the close links between gender perception and trait impressions (e.g., Mileva & Lavan, 2023).

Thus, how is a mental representation of emotional meaning formed and combined with other emerging details about the speaker (e.g., gender, age)? EEG and behavioral evidence suggests that transient (e.g., emotional state) and more stable (e.g., speaker identity and physical characteristics) nonverbal vocal aspects interact in voice perception (Berry, 1990; Hughes et al., 2004; Lavan, 2023; McAleer et al., 2014; Montepare & Zebrowitz-McArthur, 1987), potentially affecting person perception including spontaneous trait inference (Pinheiro et al., 2021). These interactions could offer a mechanistic explanation for how overgeneralization and halo effects arise (Forgas & Laham, 2022; Zebrowitz & Montepare, 2008). We often extrapolate from transient social signals (e.g., the emotional quality of the voice) to make inferences about more stable speaker characteristics (McArthur & Baron, 1983; Zebrowitz & Collins, 1997). These inferences occur automatically, within a few milliseconds of voice exposure, and even when contextual information is absent (see, for example, Freeman et al., 2020). Specifically, vocal emotion expressions of happiness, anger or fear may suggest the speakers’ capacity to act in a friendly, domineering, or submissive manner rather than simply how happy, angry or fearful they feel, respectively. This suggests, for example, that inferences that the speaker is angry (emotional cue) might not be separable from inferences that the speaker is aggressive (person characteristic), at least for unfamiliar speakers.

Several studies support links between vocal emotion expressions and trait impressions. For example, perceived emotional authenticity predicts how trustworthy the speaker is judged to be (Pinheiro et al., 2021). Research on emotional face perception reveals that people expressing happiness are typically perceived as high in affiliation, whereas people expressing anger are perceived as high in dominance (Hess et al., 2000; Krumhuber et al., 2007). A specific association between positive valence and predicted trustworthiness has also been documented in voice perception (Pinheiro et al., 2021). The emotion-trait associations may even be overgeneralized to non-emotional expressions (e.g., Freeman et al., 2020).

It is worth noting that judgements of psychological and social characteristics of speakers (e.g., trustworthiness or dominance) are not systematically affected by voice familiarity (Lavan, Kreitewolf et al., 2021; Lavan, Mileva et al., 2021), similarly to age judgements (Lavan, 2022). However, trustworthiness ratings could be affected by person knowledge. For example, in the study of Lavan and collaborators (Lavan, Kreitewolf et al., 2021; Lavan, Mileva et al., 2021), trustworthiness ratings were shaped by the valence of the vignettes listeners were provided with in an identity learning task. This suggests that, in the case of personally familiar voices, trait judgements (and, arguably, emotional judgements) could be affected by the knowledge listeners have about the speaker.

## Moving Forward

Despite promising advancements in understanding the neural and functional bases of emotional voice perception, many questions remain unanswered. The findings discussed in the previous sections suggest that the perception of emotions expressed in the voice depends not only on *how* one speaks but also on *who* speaks. This strongly motivates the need to update existing models of emotional voice perception to include more general operations related to person perception from voices. The strong interplay between emotion, identity, and person-related characteristics implies that not only emotion research should become more ‘personal’, but also that person perception research should consider the role of emotions.

This review establishes a starting point for broader research aiming to understand how the human brain translates voice acoustics into person representations that shape listener behavior and affect social interactions (Klofstad, 2016; Mileva et al., 2019; Tigue et al., 2012), particularly in determining whom we should approach or avoid. Sometimes, it is indeed *the voice that decides it all*.

## Additional Information

### Data Availability

No datasets were generated or analysed during the current study.
